# “Room to Reflect”: A Pilot Workplace Resiliency Intervention for Nurses

**DOI:** 10.3390/ijerph19127272

**Published:** 2022-06-14

**Authors:** K. Jane Muir, Jeanell Webb-Jones, Nancy Farish, Kimberley Barker, Claiborne Miller-Davis, Susan Galloway

**Affiliations:** 1 PhD Program, University of Virginia School of Nursing, Charlottesville, VA 22903, USA; 2 UVA Health: Infectious Disease Clinic, Post-Anesthesia Care Unit, Nursing Research Office, Charlottesville, VA 22903, USA; jw8dr@hscmail.mcc.virginia.edu (J.W.-J.); nancyfarish@comcast.net (N.F.); ecm9s@hscmail.mcc.virginia.edu (C.M.-D.); sjg8d@hscmail.mcc.virginia.edu (S.G.); 3 University of Virginia Health Sciences Library, Charlottesville, VA 22908, USA; krb3k@virginia.edu

**Keywords:** resilience, burnout, clinician, nurse, well-being, virtual reality, restoration, mindfulness

## Abstract

Rising workload demands for nurses necessitate the implementation of easily accessible and innovative clinician well-being resources on health care units. This pre/post pilot study sought to measure the impact of a mobile workplace intervention, “Room to Reflect” on staff nurse and nurse manager resilience. A mobile toolbox with a sound machine, Virtual Reality headset, and associated Quick Response code audio/video offerings, and a paper Pocket Guide of mindful restoration practices were provided to 7 health care units for a 3 month period. Pre/post questionnaires assessed perceived resilience using the Connor-Davidson Resilience scale, and intervention feasibility (ease of use), accessibility (spaces used), and effectiveness (restoration). Data analysis consisted of descriptive statistics, paired and independent samples *t*-tests, and Wilcoxon Signed Rank tests. From the pre (*n* = 97) to post (*n* = 57) intervention period, there was a significant difference in resilience for Clinician 3 staff nurses. A mean increase in resilience was noted among nurse managers following participation in the intervention, z = −2.03, *p* < 0.05. The Pocket Guide was the easiest offering to use, while VR offerings were accessed the most through Quick Response code. Space and time were the most common barriers to Room to Reflect use. Staff nurses felt supported by managers to use the program, and managers perceived that the program improved nurse job satisfaction.

## 1. Introduction

Clinician well-being is a critical component of health care work environments and therefore patient care delivery [1]. Establishing a culture of clinician well-being is a priority for agencies in the United States (U.S.) such as The National Academies of Sciences, Engineering, and Medicine, and the Institute for Health Care Improvement as it influences individual clinician work, interpersonal interactions (i.e., health care teams and patients) and the broader health care system [1,2,3,4,5]. Healthy work environments that support clinician well-being can directly and indirectly improve patient quality care [1].

Prior to the COVID-19 pandemic, burnout impacted approximately 30% of registered nurses (RNs) in the U.S. [1,3,6,7]. Current burnout estimates range from 30% to 55% throughout the nation and world [2,3]. The prevalence of post-traumatic stress (PTSD) among all clinicians increased during the first months of the pandemic, with particular negative impacts to RNs [4,5]. RNs experienced worsening physical and mental health as the pandemic advanced, which is concerning given their critical role in bedside patient care [6]. Increased patient volumes and acuity levels in the COVID-19 pandemic have stretched nursing workloads, which were already strained prior to the pandemic. In addition to staff RNs, clinician leaders (i.e., RN managers) have been taxed, not only in supporting the well-being of their staff/colleagues, but additionally in filling staffing gaps when necessary [7]. Overall, the COVID-19 pandemic strained clinician workloads and the ability to restore (i.e., take breaks, bring awareness to oneself) on the job. This is concerning given well-established and costly associations between decreased RN well-being (i.e., burnout, low job satisfaction, low resilience) and poor patient outcomes [1,2,4].

### 1.1. Current Well-Being Interventions

Resilience, defined as the ability to respond and adapt to a real or perceived challenge, is an important component of clinician well-being research within the context of managing job-related stress, trauma, and moral injury [8,9]. Resilience is associated with improved (i.e., reduced) burnout symptoms and reduced clinician turnover [10]. McAllister and Love (2011) identified that resilience is a skill that can be learned, and a plethora of studies identify that resilience buffers the impact of occupational stress, reduces the risk of burnout, and enhances social connections on the job [11,12,13,14]. A meta-synthesis of qualitative studies by Kim and Chang (2022) suggests that registered nurses (RNs) seek resiliency practices not only for personal well-being but professional development [15]. Such findings have positive implications for the quality of patient care delivery.

Interventions addressing clinician well-being and resilience in hospitals range from the institution of chief wellness officers, well-being rooms on units, code lavender offerings, mindfulness-based stress reduction interventions, to mandating safe/sufficient RN staffing ratios as a means to reduce burnout [1,16,17,18,19,20,21]. A meta-analysis of 13 RN resilience intervention studies consisting of mind-body exercises, in-person discussions, and web-based programs by Zhai and colleagues (2021) found improvements in resilience, stress, anxiety, and depression compared to RNs who did not participate [22]. Mindfulness, the act of paying attention to thoughts, sensations, personal and interpersonal interactions in the moment in a non-judgmental way, is an accessible well-being intervention commonly implemented in health care work environments [23]. Mindfulness-based stress reduction interventions are practices that cultivate awareness and compassion through practices ranging from breathing meditations, walking practices, journaling, and dyad work [23,24,25]. A pilot mindfulness and self-compassion program, ‘Replenish at Work’, provided clinicians with short restorative practices with 65% of participants reporting improved ability to manage occupational stressors such as patient care delivery [26]. Finally, an on-the-unit pilot intervention for pediatric intensive care unit RNs delivering brief meditations before shifts reduced reported stress in the pre- to post-intervention period [27].

Although such interventions have demonstrated efficacy in reducing burnout, perceived stress and/or increasing resiliency, they often are time-consuming, requiring clinicians to participate in activities after their work shifts or external to the health care unit. For example, mindfulness-based stress reduction interventions have demonstrated improvements in clinician resiliency and reduced stress but often require clinicians to leave their immediate work setting to participate [19,28,29]. Such limitations restrict a participant’s use of interventions and ability to transfer such practices into the work setting. An additional cultural limitation of current well-being interventions is the expectation that individual RNs alone are responsible for their self-care, while, as Udod and colleagues (2021) asserted, the resilience of a health care organization is a shared responsibility between RNs, unit leadership, and systemic leadership [30]. More interventions overall are needed that integrate unit leadership to support RN well-being. 

### 1.2. Gaps in Well-Being Interventions

Recognized limitations of resiliency-building activities in the workplace have contributed to an increase in accessible innovative tools and interventions in the health care setting. Mintz-Binder and colleagues (2021) assessed the effectiveness of a pilot resiliency toolkit with lavender aromatherapy, phone application games, crossword puzzles, and instructional cards about meditation and deep breathing for RNs in four urban hospitals in the Southeastern U.S. [31,32]. RNs using the toolkit experienced decreased perceived stress at work and increased resilience scores. In a study replicating this initial pilot work, Andersen and colleagues (2021) found that participants most frequently used the breathing exercise cards, lavender aromatherapy sticks, and coloring books on the unit [32]. Such research demonstrates that easily accessible and innovative resiliency tools are necessary to support RNs’ work. 

Beyond the use of toolkits, a range of technological devices that are mobile, space-saving, and effective in enhancing well-being have emerged as innovative methods of restoration and relaxation. One such device is the Virtual Reality (VR) Optical Head-Mounted Display, a device that allows the user to experience a convincing computer-generated simulation, complete with auditory and visual elements [33]. Optical Head-Mounted Displays are also used to access 360° videos, which are created by filming an environment with cameras equipped with multiple fisheye lenses to produce a spherical image [33]. The result is a video that captures a 360° view of the setting, and which is controlled by the viewer, allowing them to change perspective simply by turning their heads. In VR simulations, users can interact with and change the computer-generated environment in ways that are appropriate to the setting, (e.g., shooting a bow and arrow in an archery game or causing stands of seaweed to sway in response to a hand movement in an underwater simulation). In a 360° video, however, the viewer cannot interact with the environment beyond the ability to control her own body, turning to look up, down, and in a complete circle.

VR and 360° video have been identified as methods for reducing anxiety and stress by bringing individuals to a relaxed mental state through immersion in a relaxing simulation (e.g., nature landscapes, meditation) [34,35]. Devices transmitting relaxing sounds, such as sound machines programmed with lower frequency “brown” noises (such as heavy rainfall and rumbling thunder), “pink” noises (such as moderate rainfall and ocean waves), or low frequency music, have shown to reduce anxiety and depressive symptoms, as well as sleep difficulties [36,37].

### 1.3. Addressing the Gap

Few studies to date integrate mindfulness practices with innovative technology such as VR for clinician well-being interventions. The innovative work from Mintz-Binder and colleagues (2021) indicated that easily accessible restorative practices are beneficial for RNs and suggested that other tools such as VR, sound machines, and phone applications may further support clinician restoration [31,32]. Given the high demand for interventions supporting clinician work that are easily accessible immediately and in the work setting, we sought to develop and measure a program using innovative technologies for clinician restoration.

The purpose of this pre/post quasi-experimental study was to describe the feasibility, accessibility, and effectiveness of a well-being intervention, “Room to Reflect” (R2R) among RN staff and RN managers. The specific aims of the study were to (1) measure RN resilience before and after the R2R intervention and (2) assess R2R feasibility (frequency of use, barriers to use), accessibility (space, support for use), and effectiveness (relaxation, restoration, ease of use) of R2R. A tertiary sub-aim of the first study aim was to assess RN manager perceptions of intervention impact, success, and barriers on their units given established associations between leadership empowerment and reduced RN burnout and stress [38]. 

## 2. Conceptual Overview and Context of Intervention

This study was guided by the concept of *job design* which focuses on the context in which people work [39]. Job design captures the processes and outcomes of work, as well as the structure and enactment of work [38]. This conceptual guide influenced the study purpose and implementation as a means to address an issue important to health care workplace structure, context, and work delivery: RN coping with in-the-moment job stressors.

It should be noted that the current study represents just one of many interventions necessary to address the issue of clinician stressors. This study acknowledges existing conceptual perspectives around job demand and workplace efficiency. Health care delivery has transformed to meet patient demands in an efficient, fast-paced method. This idea can be likened to Hartmut Rosa’s concept of social acceleration, or the “picking up of the general pace of life” (p. 3, [40]). Similar to social acceleration, the increase in a culture of health care efficiency is driven in part by technologic advancement. The proposed intervention represents an “intentional form of social deceleration” (p. 15), in which individual-level strategies aim to remove individuals, at least temporarily, from the fast-paced, high-stakes nature of the work environment [39]. It is important to note the a priori limitations to this approach, specifically that (1) they do not represent a systems-level well-being intervention directly; (2) they may reinforce a culture of efficiency in the health care workplace; (3) the approach is one specific intervention that does not represent the ideal tool for well-being/restoration for all people. 

Rather, the current study represents just one approach to a form of occupational health promotion aiming to reduce or aid in the “negative aspects of well-being (e.g., stress)” (p. 4, [41]). The study also fills a conceptual gap identified by Tetrick and Winslow (2015) related to understanding which modalities of workplace coping interventions are most beneficial for employees/workers [40]. This study was developed at the onset of the COVID-19 pandemic, where resources for well-being restoration—specifically break rooms—were eliminated or renovated for patient care delivery or personal protective equipment storage.

## 3. Materials and Methods

### 3.1. Study Design and Aims

This pilot intervention study, “Room to Reflect” (R2R), used a basic pre/post design to assess resilience, program feasibility, accessibility, and effectiveness among RNs after using a mobile toolbox and Pocket Guide as on the job well-being resources. The study took place at a single academic medical center over a 3 month period across 7 units split into two cycles from June to November 2021. Each cycle started with a 30-day enrollment period where participants were consented, educated about how to use R2R, and filled out the pre-intervention questionnaire. Participants then used the R2R offerings for 2 months. The first cycle consisted of 3 units, while the second cycle had 4 units. Participating health care units were located within inpatient, outpatient, pre-operative, and post-operative settings.

### 3.2. Room to Reflect Intervention 

**Figure 1:**
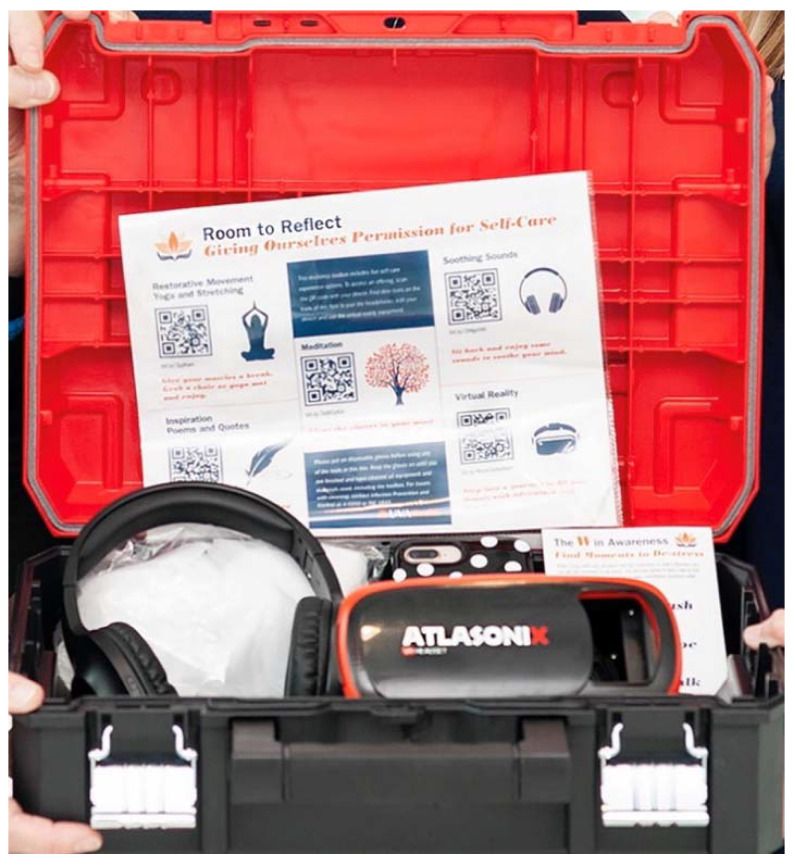
Mobile Toolbox with technology contents, Pocket Guide, and poster offerings.

**Figure 2:**
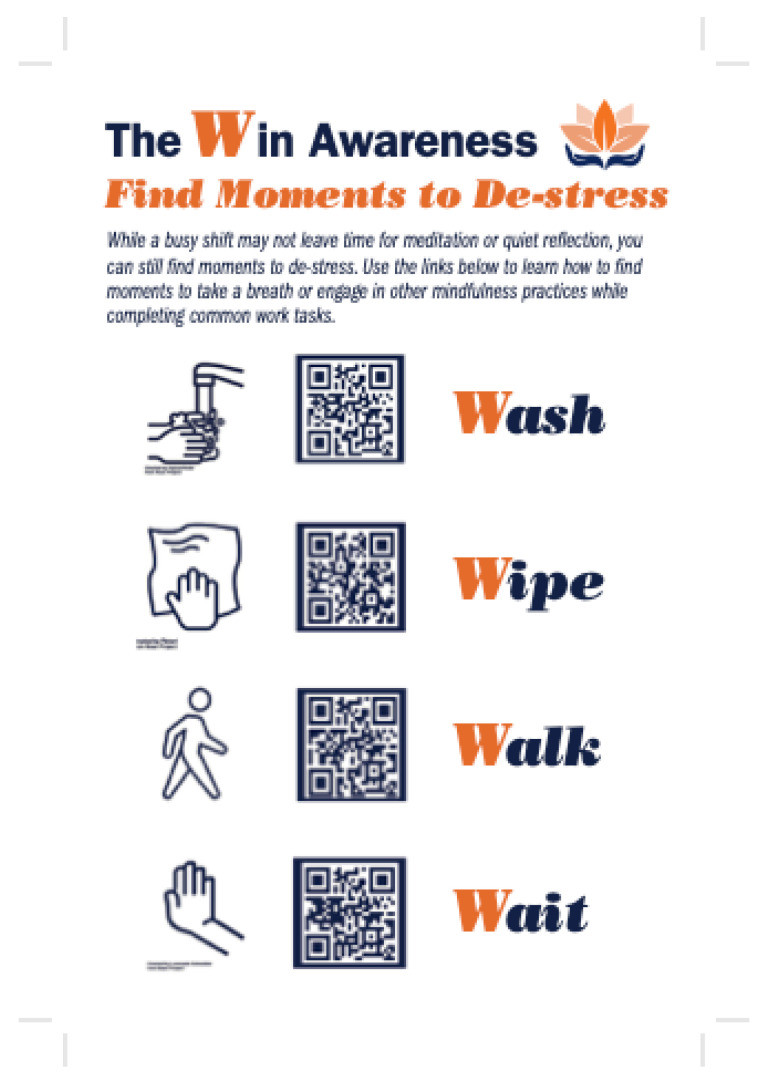
Pocket Guide with Quick Response Codes.

The R2R program consisted of two elements: (1) a mobile toolbox and (2) Pocket Guide. The mobile toolbox (Figure 1) was a physical toolbox container with a wide variety of technological offerings that could be taken to any available space as time allowed. The Pocket Guide (Figure 2) was a paper card that provided participants with written and pictorial representations of mindfulness-based “in the moment” practices at work.

One toolbox with contents were provided for each pilot study unit (7 units, 7 toolboxes). Study participants on each unit utilized their personal mobile devices to access the offerings located within the toolbox. The toolboxes were not accessed in a group setting. All mindfulness practices were done individually.

#### 3.2.1. Mobile Toolbox

**Figure 3:**
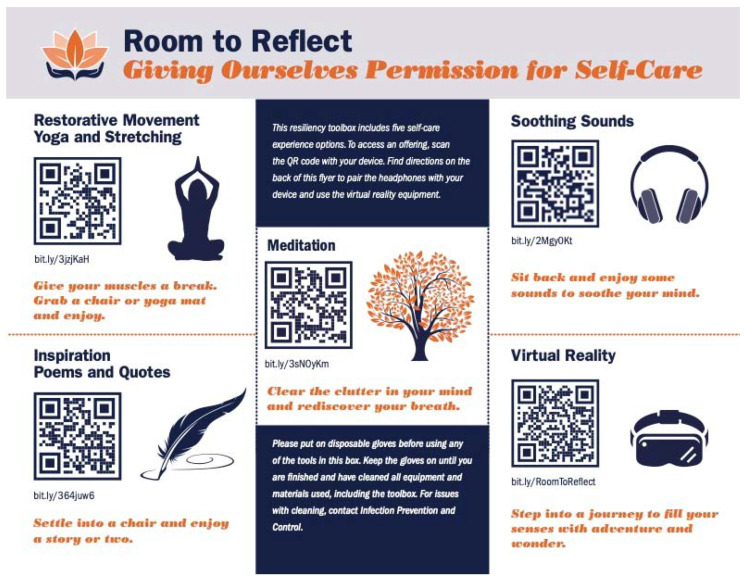
Poster Offerings with Quick Response Codes.

The mobile toolbox contained an Optical Head-Mounted Display for Virtual Reality (VR), wireless Bluetooth headphones, and a sound machine. A laminated poster (Figure 3) with a list of mobile restorative offerings was included in each toolbox with associated Quick Response (QR) codes for participant access. There were 5 categories of offerings and associated QR codes on the poster: “Restorative Movement with Yoga and Stretching”, “Soothing Sounds”, “Inspiration Poems and Quotes”, “Meditation”, and “Virtual Reality”. Each category had a picture associated with the activity, a brief invitation about the offering and the QR code for mobile device access. After scanning the QR codes, participants were led to a SoundCloud or YouTube page to access the audio/video offerings discussed as follows. 

#### 3.2.2. Quick Response Offerings: Audio and Visual

An overview of each offering is provided in Table 1. Briefly, “Restorative Movement with Yoga and Stretching” contained curated YouTube videos that participants could watch or listen to after scanning the QR code for accessible chair yoga and stretching practices. “Soothing Sounds” contained audio files on SoundCloud with relaxing nature sounds. “Inspiration Poems and Quotes” contained audio files of poetry and quote reading. “Meditation” contained audio mindfulness-based meditation and awareness practices. Finally, “Virtual Reality” contained YouTube videos that participants could watch with the Optical Head-Mounted Display across a variety of categories: travel, nature, and animals. Each of the offerings from the poster were either curated by the study co-investigators or were filmed by faculty/staff at the study institution. Of note, given that the term “VR” was in more common use at the time of the study than the term “360° video”, and with the understanding that the immersive nature of 360° videos offers similar mental wellness benefits as VR, the term “VR” is referenced throughout the study instead of the more technically accurate “360° video”. 

#### 3.2.3. Pocket Guide

The Pocket Guide (Table 2) was created as an alternative method to the mobile toolbox to engage in mindfulness and restorative practices when stepping away from the bedside was not feasible. The study team identified four common daily tasks that all clinicians engage in while at work and synthesized these observations with established mindfulness-based practices (e.g., body scan meditation) to create, ‘The four Ws of awareness’. The Ws included ‘Walk’ (e.g., in and out of patient rooms), ‘Wipe’ (e.g., wiping down equipment), ‘Wash’ (e.g., washing of the hands), ‘Wait’ (e.g., on the phone giving a handoff report) [23]. The activities were represented on a 3 × 5 laminated card for each participant to carry while working. Each ‘W’ on the card was represented with the word and a picture of the activity along with an associated QR code to scan with a mobile device. Once accessed, the participant was given a list of prompts and examples associated with the particular activity with restorative and mindfulness practices to engage in. Participants were given a Pocket Guide at the start of the study and it was also provided in the mobile toolbox. 

#### 3.2.4. R2R Offering Development

A significant component of the project was the study team engaging frontline RNs in developing the study intervention prior to the study. For example, to create the unique audio and visual (e.g., VR) toolbox offerings, the study team informally asked nursing colleagues what sort of sounds help them relax. VR categories were sourced from staff experiences with hobbies and places they know to be relaxing.

### 3.3. Sample

A convenience sample of staff RNs and RN managers across 7 units were recruited across medical-surgical, post-anesthesia care, infusion outpatient, pediatric acute care, transplant, and preoperative preparation units. Inclusion criteria consisted of staff RNs and managers with at least three months of experience, and age 18 years or older with the ability to consent. 

Study recruitment first consisted of announcing the program at hospital-wide manager meetings. Announcements were then made at individual unit huddles about the program. Interested participants were consented for the study and were provided a unique identification (ID) number for survey completion. Ethical approval for the study was obtained by the Institutional Review Board (IRB HSR# 22987). 

### 3.4. Study Procedures

#### 3.4.1. Questionnaire Dissemination

All participants were provided a QR code to access a pre-intervention questionnaire consisting of demographic questions and the Connor Davidson Resilience Scale (CD-RISC 10) survey [50]. At post-intervention, RN staff completed a questionnaire with the CD-RISC 10 and questions about R2R feasibility, accessibility, and effectiveness. The RN managers’ questionnaire only included the CD-RISC 10 and manager-specific questions about R2R implementation. 

#### 3.4.2. Research Assistant Role

Throughout the study period, research assistants taught study participants how to access R2R offerings (i.e., VR Optical Head-Mounted Display, sound-eliminating headphones). Throughout the study period, the RAs visited the unit clinics weekly during daily huddles and/or staff meetings to follow-up with participants. The RAs were a resource to the participants and available if they had questions about the offerings. 

### 3.5. Variables and Questionnaire Tools

Study variables assessed resiliency (measured by CD-RISC 10), and R2R program feasibility, accessibility, and effectiveness. Questions pertaining to the study measures were created using Qualtrics Software which participants accessed through a QR code in the pre- and post-intervention period. Questionnaires consisted of Likert-style, yes/no, and free-text response questions. Sample questions are found in Table 3.

Bit.ly, a freemium URL-shortening software, was employed as a usage statistics gathering tool: a unique shortened URL was created as a link redirect for each offering in the toolkit: one for each “Ws” QR code in the Pocket Guide, one for the QR code linked to the SoundCloud offerings, and one for the QR code linked to the R2R YouTube channel, which was created to host the 360° videos playlists. This allowed the research team to monitor which category of offerings were the most clicked on by participants and, based on that information, determine which of the category offerings should be expanded. 

#### 3.5.1. Demographics

Demographic characteristics collected in the pre-intervention study period included age, race, ethnicity (Collection of ethnicity information is considered standard demographic inquiry as dictated by the United States Office of Management of Budget, referencing “Hispanic or Latino” and “Not Hispanic or Latino” [51]), years of clinical practice, and clinical ladder level (i.e., professional ladder classification by Clinician 1 to Clinician 4). The Clinical Ladder designation pertains to the level of professional achievement of the clinician based on a variety of institutional factors (e.g., years in the hospital, leadership involvement, mentorship, research, etc.). For example, a new RN on the health care unit at this specific institution is considered a Clinician 1, while often (not always), a more senior RN could be a Clinician 4. Demographics were completed by all participants.

#### 3.5.2. Connor Davidson Resilience Scale 10

Resilience was measured using the Connor Davidson Scale (CD-RISC 10), a 10-item scale validated globally and across a plethora of populations, including nurses with a test-retest correlation of 0.87 [52,53,54]. The CD-RISC 10 is a 10-item validated survey (reliability α > 0.86) that assesses the ability to adapt to change and stress, as well as the ability to remain focused, think clearly, and manage unpleasant feelings such as pain, anger, and sadness. The survey consists of a series of statements that participants answer on a Likert scale ranging from 0 (not true at all) to 4 (true nearly all of the time). Total scores range from 0 to 40 with higher scores indicating increased ability to cope with stressors and adversity [40]. 

#### 3.5.3. Feasibility

All participants were asked to report how frequently they accessed the R2R offerings, ranging from “none” to “21+ times”. Additionally, all participants were asked to select which barriers prevented them from using R2R (e.g., lack of time, physical space limitations, etc.). 

#### 3.5.4. Accessibility

Appropriateness of space was assessed among all participants (yes or no), as well as why or why not by using free-text response. Additionally, participants were asked if they perceived that leadership and co-workers on the unit supported the use of R2R on the unit. 

#### 3.5.5. Effectiveness

Participants were asked dichotomously (i.e., yes or no) if resources within the R2R toolbox offered relaxation and restoration. Participants were also asked to assess ease of use (e.g., time efficient, motivation to use) of all R2R resources ranging from “very easy” to “not used”. 

#### 3.5.6. RN Manager Evaluation

In addition to the aforementioned survey questions, RN managers completed questions relevant to perceived effectiveness of program implementation. Managers were asked to specify how they supported their bedside RNs, and what barriers, if any, prevented them from supporting staff. 

### 3.6. Data Analysis

Statistical analyses were performed using IBM Statistical Package for Social Sciences, version 28 [55]. Prior to analysis, all variables were checked for data entry errors and missing values. Subjects were excluded listwise if missing either a CD-RISC 10 pre-/post-intervention response. Demographics and the R2R Program Evaluation Survey were analyzed using descriptives and frequencies for all participants. Spearman’s rank correlation was computed to assess the relationship between pre- and post-intervention resilience scores using CD-RISC, as well as age, gender, and clinical ladder level at a significance level of *p* < 0.05 and <0.01. 

CD-RISC scores were tested by paired samples *t*-test for RN pre- and post-intervention times, as well as independent samples *t*-tests. As the RN manager sample size was small and violated other assumptions for parametric tests, the non-parametric statistic Wilcoxon Signed Rank test was conducted. Free-text responses were analyzed on a content level and by frequency of response. Specifically, free-text responses were classified based on category of assessment (i.e., barrier to use, space issues, lack of time) and enumerated by frequency of response. 

## 4. Results

There were initially 97 participants enrolled in the study including 89 staff RNs and 8 RN managers. After dropouts and exclusion of participants who did not complete both pre- and post-intervention questionnaires, a final sample size of 57 was obtained with 50 RNs and 7 RN managers. Complete demographic information can be found in Table 4. Spearman’s rank correlation findings are outlined in Table 5. 

### 4.1. Effectiveness: Resilience

CD-RISC means are outlined in Table 6 and Table 7. The median (Md) CD-RISC score for RNs in the pre-intervention period was 29; the median for RN managers was 28.5; the median overall was 29. At post-intervention, the median at all levels was 30. These values align with study means reported in Table 6 and Table 7. Among staff RNs, there was no statistically significant increase between pre-intervention *(M* = 28.9, *SD* = 4.8) to post-intervention scores (*M* = 29.7, *SD* = 5.0), t (49) = 1.32, *p* > 0.05. There were no statistically significant differences in resilience scores among the RN and RN manager groups between the pre-intervention and post-intervention period (Table 6 and Table 7). 

Amongst staff RNs, there was only one (1) Clinician 1 who participated in the study, but there were 28 Clinician 2, 14 Clinician 3, and 6 Clinician 4 RNs. A paired sample *t*-test revealed a significant difference between pre-intervention (*M* = 29.6, *SD* = 4.7) and post-intervention scores (*M* = 31.6, *SD* = 37), t(13) = −2.3, *p* < 0.05 for Clinician 3 RNs. The eta square statistic (.27) indicated a small effect from the intervention. 

A Wilcoxon Signed Rank test revealed a statistically significant increase in CD-RISC scores for RN managers following participation in the intervention, z = −2.4, *p* < 0.05, with a large effect size (r = 0.51). The median score on the CD-RISC survey increased from pre-intervention (Md = 28.5) to post-intervention (Md = 30.0). 

### 4.2. Feasibility: Program Use and Barriers

The majority of participants (47.4%) accessed the offerings at least once over the 3 month study period, with the greatest frequency of use being 1 to 5 times. The most common barrier to accessing the R2R resources reported among most participants (72.9%) was lack of time, followed by inability to easily access a quiet, private space (33.9%). When participants could access a space for R2R use, participants felt that the allocated space for use was appropriate (50.9%) (Table 8). 

### 4.3. Accessibility: Space and Support for Use

In free-text responses, participants stated that they used R2R most commonly in designated quiet/restoration rooms or unoccupied patient rooms on the unit (50.2%). Participants who felt that the designated space for R2R use was not ideal attributed this to a lack of break room/quiet space (33.3%). Participants reported having leadership and co-worker support to use the R2R offerings (66.7%) (Table 8). 

### 4.4. Restoration/Relaxation and Ease of Use

Among participants who used the offerings, the majority of participants (42.1%) reported that the Pocket Guide, followed by “Soothing Sounds” audio (31.2%) provided the greatest relaxation and restoration (Table 8). The Pocket Guide was the easiest to use (40.4%) (Table 9). 

The usage data (Table 10) collected via the unique Bit.ly links embedded in each category’s specific QR code reflects that the VR/360° videos were the most accessed of all the offerings (440 clicks) over the study’s three-month period. Of the YouTube channel’s five VR playlists, “Experience Animals” was the most popular (154 clicks, or 35%). According to the Bit.ly usage data, the audio resources hosted on SoundCloud were the third most popular set of offerings (125 clicks), with the Meditation offering garnering the most clicks (57, or 45.6%). 

### 4.5. RN Manager Evaluation

Among RN managers, the majority (75%) reported that the project was successfully implemented on their unit (Table 8). The most common method for support staff to use R2R by managers was encouraging staff verbally to take the time for themselves (85.7%). Managers attributed high patient acuity as a significant barrier to supporting staff (40.1%). Managers reported that the implementation of R2R on their unit most impacted staff’s ability to handle stress and staff job satisfaction. Lack of time was a reported free-text response among managers as a barrier to participant use. 

## 5. Discussion

The “Room to Reflect” (R2R) intervention sought to provide RNs with accessible, in the moment restorative practices that integrate mobile technologies. Over 58% of study participants were retained from the pre- to post-intervention period across a range of clinical experience years (e.g., Clinician 1, Clinician 2, etc.). The study was feasible in that the majority (47.4%) of participants accessed at least one offering from R2R over the study period, although the frequency of offering use was 1 to 5 times over a 3 month period. This low frequency of offerings may be attributed to the lack of time and space available for RNs to use the offerings, as participants reported in study accessibility questionnaires. Participants reported that the Pocket Guide was the easiest resource to use in R2R and resulted in the greatest perceived relaxation and restoration across offerings. The VR offering was the most accessed through QR scan. The majority of RN managers (75%) felt that the program was successfully implemented on their units. RN managers felt that the program improved staff RN satisfaction and job stress. 

Resiliency scores during the study, as measured by the CD-RISC 10 were in the bottom (0–29) and middle (30–32) quartiles [50]. There was no significant change in mean resiliency scores as measured by the CD-RISC 10 among staff RNs, nor among staff and manager RNs when evaluated in aggregate. There was a significant increase in mean CD-RISC scores among Clinician 3 RNs compared to Clinician 1, 2, and 4 RNs (i.e., the clinical ladder). Finally, there was a mean increase in CD-RISC scores among RN managers as revealed by the Wilcoxon Signed Rank test. It is possible that increased experience of Clinician 3 RNs comparable to their colleagues may have contributed to improvements in CD-RISC scores across the study period, given data supported by Kelly and colleagues (2021) demonstrating associations between RN tenure and increased resilience [56]. However, a higher clinical ladder designation does not necessarily indicate increased experience/tenure on a unit but rather increased involvement in improving one’s health care unit and hospital from a service (i.e., precepting, research) perspective. Increases in CD-RISC scores among RN managers from the R2R intervention in our study is supported by Carter and Turner’s (2021) study evaluating RN leader resilience after a targeted mobile intervention [57]. Similar to our study findings, RN manager resilience increased after online resilience-building activities applied in the clinical context [57]. Our findings suggest that RN leaders are in great need of well-being interventions as they not only manage patients and logistics of health care units, but also care for their RN staff [58].

Participants in the R2R program reported that the Pocket Guide was the easiest offering to use. Our Pocket Guide was a 3 × 5 inch card with pictorial representations of mindful practices that participants could engage in for building awareness. The Pocket Guide’s pictorial representations of mindful activities (e.g., hands “washing”) may have aided in reminding participants how to restore in a more practical and accessible way comparable to other offerings. Its increased use may also suggest that more time and space for restoration, as well as education, is needed for participants to use the VR and sound machine offerings. This finding aligns with Mintz-Binder and colleagues’ (2021) toolkit intervention, where paper resources were a highly accessed offering for restoration [31,32].

An intriguing finding from our usage data was that the VR offerings were the most commonly accessed QR codes. Given that our usage data did not explore how long or which participants accessed the offering, we cannot specifically extrapolate if each QR scan meant that participants were using the offering. In other words, high usage from this QR code may have been attributed to participant experimentation instead of offering usage (i.e., scanning the QR code but not actually participating in the offering). It is also important to note that the Pocket Guide, which had a QR code associated with it, was the second accessed QR code, despite participants rating it the easiest to use. This may be due to the fact that the Pocket Guide did not necessarily require scanning the QR code for use. Participants could simply slip the guide into their pocket and look at the visual depictions for access to mindfulness resources as opposed to scanning the QR codes on the guide for use. Caution must be used in interpreting this data, particularly as most participants reported that the Pocket Guide (with 285 clicks according to Bit.ly) was the most useful of all the resources which were offered. The high usage statistics around the R2R YouTube channel could indicate one-time curiosity on the part of participants who, ultimately, found little value in the 360° videos and did not access them repeatedly.

Our findings are distinct from published resiliency intervention pilot studies in that we recruited unit managers for participation in addition to frontline RNs. This inclusion of unit administrators facilitated an understanding of leadership support and perceived barriers for implementing resiliency projects on the unit. Including leaders in this pilot initiative was critical for RNs to feel that they can restore at work, particularly during the burdensome COVID-19 pandemic. Overall, our findings are supported by Mintz-Binder and colleagues’ (2021) inference that unit RNs are drawn to use resiliency practices when they are encouraged by leadership to do so [31,32].

Our study findings have important implications for clinician well-being interventions. First, despite our program promoting mobile, easy-to-access tools, RNs still encountered challenges with time and space to use R2R. Such findings could derive from a variety of factors such as: (1) inadequate staffing on units to support program use; (2) cultural barriers preventing RNs to seek restoration/relaxation (e.g., an aversion to leaving team members/patients to care for oneself); (3) the absence of dedicated spaces for restoration/relaxation, among other factors, and (4) increased workload demands due to the COVID-19 pandemic [59,60,61]. The aforementioned “aversion” to self-care in the workplace parallels Sonnentag’s “Recovery Paradox” (p. 173) which posits that people with a high level of job stressors have a tendency *not* to recover/restore when necessary [62]. Relatedly, and specific to nursing, Steege and Rainbow (2017) identify the “Supernurse” (p. 20) culture wherein RN values of caring for all (patients and colleagues) and not showing weakness in the workplace serve as barriers to RNs coping with workplace fatigue [63].

Despite these challenges, the majority of participants perceived that unit leadership supported the R2R program, which is often a critical barrier to implementing well-being programs from a cultural and financial perspective [32,38,64]. Finally, participants identified that the Pocket Guide was the most accessible offering. Although this finding supports the implementation of well-being offerings that can be simply placed in an RN’s scrubs, it supports our other findings that time and space constraints limit self-care opportunities. More resources are needed on health care units to provide clinicians with space and time for restoration in order to adequately engage in patient care.

### 5.1. Limitations

A significant limitation of this study was the lack of a control group. Across the plethora of mindfulness-based and recovery interventions aimed at improving clinician coping in the workplace, this aspect of the study design is an important component of understanding intervention effectiveness [41]. While a significant component of this study was aimed at understanding project feasibility and accessibility of the technologic features of R2R, including a control group will be vital for future study applications. Additionally, this study experienced a high number of dropouts from the pre- to post-intervention period. Part of this attrition may be attributed to the project taking place during the COVID-19 pandemic with a high-level of job unpredictability on health care units.

Our intervention was limited to a single academic medical center. Despite this limitation, our sample included RNs from a range of health care units, which offered a unique perspective on R2R given the variability in patient acuity levels and volumes across the units. Our study also represented only one of a multi-faceted approach needed to transform health care work environments beyond individual-level coping tools. A potential source of bias in this study was the inclusion of RN leaders as a secondary sample. In other words, bedside RNs may have felt biased to report positively about their experiences in R2R [65]. To address the limitations, we appointed study coordinators to visit the enrolled units at least twice weekly to communicate directly with bedside RNs, understand concerns, and address any potential conflicts of interest with leadership. Finally, our study was implemented during the COVID-19 pandemic, which presented unprecedented uncertainty with participant engagement given the daily uncertainty of patient demands. We actively worked to address this limitation by having study coordinators visit the health care units frequently to interface with participants.

An additional limitation with our reporting of access using the QR codes (i.e., YouTube) was that usage tracking on an individual level was not accessible. Rather, reporting was conducted only on an aggregate level over the course of the study period. Such limitation pertains to YouTube capabilities and the use of Bit.ly. Future studies would integrate a method to eliminate this barrier to reporting.

Methodologically, our study was largely limited to descriptive analyses of program resiliency impacts, feasibility, accessibility, and effectiveness. As a result, we were unable to draw comparisons across groups beyond mean resiliency scores, limiting an analysis of program characteristics that may be associated with influencing resiliency. Finally, there was participant variability in responses (i.e., response rate) for the feasibility, accessibility, and effectiveness questions.

### 5.2. Future Studies

Our study establishes the necessary groundwork for integrating mobile technologies into the health care space for well-being. Our findings establish that RNs are interested in and can use technologies that efficiently help them restore in the workplace. Future studies can replicate our study findings on a larger scale and across clinician groups, with robust study designs (i.e., randomized controlled trials). Evaluating the cost-effectiveness of well-being interventions is also an important component of future related research as hospitals look to financially invest in viable options for clinician retention. Understanding cost savings and benefits associated with increasing RN resilience is critical for organizational investment in RN retention and high-quality patient care.

Future applications of R2R in research must include important aspects of job context, given that RN work is in no way homogenous across health care units, despite many similarities in aspects of nursing work [66]. Identifying approaches to evaluate the effectiveness of R2R related to job context can inform aspects of job control that differ across health care settings [66]. The importance of identifying intervention effectiveness across different groups of clinicians and different settings can critically inform systems-level approaches to transform the health care work environment and clinician well-being.

## 6. Conclusions

The “Room to Reflect” program was an accessible, technology-based intervention aimed to support RN resilience in the workplace. No differences were found in mean resiliency scores in the pre- to post-intervention period among the combined RNs and RN manager groups, however, Clinician 3 RNs and RN managers experienced mean increases in resiliency scores (as measured by CD-RISC). The Pocket Guide was the most accessible and restorative offering in the R2R program. RNs reported support from RN managers to use the offerings on their shifts, and RN managers perceived that the R2R program improved RN stress management and job satisfaction. Overall, the study was feasible and effective, and highlights the importance of addressing systemic time and space-related barriers to RNs in accessing restoration at work. The findings from this program establish a groundwork for future development of well-being interventions integrating mobile and easy-to-use well-being technologies.

## Figures and Tables

**Table 1:** Room to Reflect Toolbox Offerings.

Toolbox Offerings	Rationale for Selection	Description
Restorative Movement Yoga and Stretching	Associated with reductions in health care worker stress and anxiety; improvements in self-regulation and self-compassion [42,43].	Short guided video exercises in chair yoga, gentle stretches, and mindful movement. Participants used mobile devices and noise-canceling headphones.
Inspirational Poems and Quotes	Interventions with poetry readings associated with increased empathy for families, co-workers, and patients [44,45].	Written and recorded short stories/poems. Participants access offerings with their mobile devices and noise-canceling headphones.
Meditation	Associated with reductions in perceived stress and burnout, as well as increased compassion.	Brief guided exercises on paying attention to the body and breathing. Participants listen with noise-canceling headphones.
Soothing Sounds	Sounds considered to be calming with low frequency are associated with improvements in sleep, quality of life, and depression [46,47].	Curated sounds of nature from rain, to birds, to the roaring river rapids. Participants listened with noise-canceling headphones.
Virtual Reality	Curated landscapes emanating calming scenes associated with decreases in anxiety and stress and increases in social connectedness [25,33,34,35,48,49].	VR ^1^ goggles compatible with mobile devices. Participants used their mobile devices to engage in 3D ^2^ visual experiences including animals, nature and art.
Sound Machine	Transmission of low frequency noises or calming sounds associated with reductions in anxiety, depressive symptoms, and sleep difficulty [36,37,46,47].	Compact and portable with a timer; a variety of soothing sounds of nature to provide a relaxing and peaceful experience.
Pocket Guide	Easily accessible self-care practices for physical use and reading associated with stress reduction and increased resiliency [31,32].	3 × 5 laminated card stamped with QR ^3^ codes associated with pictorial representations of four everyday activities (e.g., Wash, Wipe, Walk, and Wait) conducive to mindfulness practices.

^1^ Virtual Reality, ^2^ Three-dimensional, ^3^ Quick Response.

**Table 2:** The Ws of Awareness: Quick Response Nurse Pocket Guide *.

“W” Practice	Description
**Wash**	Prompts included 3 suggestions: (1) A simple breathing technique while approaching the sink (2) Awareness of senses while washing the hands (sight, hearing, touch) (3) Awareness of thoughts
**Wipe**	Prompts include the idea of transition from one task to the next with 2 suggestions: (1) Awareness of thoughts with prompts on how to identify and let go (2) A simple breathing technique to focus on the present moment and sensations.
**Walk**	Prompts include 4 suggestions: (1) Simple focus on the physical aspect of walking and paying attention to thoughts (2) Shorter walk prompt: Focusing on the breath while walking (3) Longer walk prompt: Focusing on all the senses (4) Practice of non-judgment of thoughts as “clouds in the sky”
**Wait**	Prompts identify examples of situations where waiting may occur during a busy day along with suggestions: (1) Simple breathing techniques with each encounter (2) Identifying thoughts (3) Present moment practice of using sensations (smell, touch, hearing)

* Prompts informed by mindfulness and compassion texts/references [23,24].

**Table 3:** Surveys and Sample Questions.

Survey	Format	Description	Sample Question
CD-RISC ^1^ Score	Likert-style questions	Measures perceived resilience	I am able to adapt to change.
Demographics	Single-select answers	Assesses sample characteristics	How many years of clinical experience do you have?
Frequency of Access	Single-select answer	Amount of times R2R ^2^ was accessed	How many times did you access the R2R program offerings?
Barriers to Access	Select all that apply; free-text	Factors that prevented access to R2R	What barriers did you encounter that prevented R2R use?
Restoration/Relaxation	Dichotomous yes/no	Perceptions of effectiveness in feeling restored using R2R	Did you experience feeling relaxed and restored using Virtual Reality?
Ease of Program Access	Likert-style questions	Assesses level of ease accessing aspects of program	How easy was the Virtual Reality to use?
Appropriateness of Space	Dichotomous yes/no	Assesses quality of space to use R2R	Did you feel that the space for R2R was appropriate?
Support for Program	Dichotomous yes/no	Assesses perceived support use R2R	Did you have leadership/co-worker support for R2R?
Impact of Program ^3^	Dichotomous yes/no; select all that apply	Assesses managers’ perceived impact of R2R on unit	Did you feel that R2R had an impact on your unit?
Supporting Program ^3^	Select all that apply	Assess managers’ ability to support staff	How did you support R2R on your unit?
Implementation of Program, Barriers ^3^	Select all that apply	Assess managers’ perceived barriers	What barriers did you find in implementing R2R on your unit?

^1^ Connor Davidson Resilience Scale; ^2^ Room to Reflect; ^3^ RN managers’ question.

**Table 4:** Participant Demographics (*n* = 97).

Characteristics	Count	%
**Clinical Ladder**		
Clinician 1	3	3.1
Clinician 2	53	55.2
Clinician 3	24	25.0
Clinician 4	8	8.3
Other, no specification	1	0.2
Manager/Assistant Manager	8	8.2
**Length of RN ^1^ practice**		
Less than 1 year	3	3.
1–5	29	29.9
6–10	18	18.6
11–15	13	13.4
Greater than 15	34	34.1
**Race**	Count	
Asian/Asian American	3	3.1
Black/African American/African/Caribbean	7	7.2
Hispanic/Latinx	1	1.0
White/European/Middle East/North African	84	86.6
Other	2	2.1
**Ethnicity**		
Hispanic	3	3.2
Non-Hispanic	91	96.8
**Gender**		
Male	12	12.4
Female	85	87.6
**Age**		
20–30	24	24.7
31–40	27	27.8
41–50	21	21.7
51–60	18	18.6
61+	7	7.2

^1^ Registered Nurse.

**Table 5:** Correlations between Resilience Scale, Gender, Age, Clinical Ladder.

Pre-Intervention				
Variable	1. Resilience	2. Gender	3. Age	4. Clinical Ladder
1. Resilience Scale	-	−0.08	0.26 **	0.10
2. Gender	−0.08	-	0.08	0.27 *
3. Age	0.26 **	0.08	-	0.22 *
4. Clinical Ladder	0.10	0.27 *	0.22 *	-
Post-Intervention				
Variable	1. Resilience	2. Gender	3. Age	4. Clinical Ladder
1. Resilience Scale	-	−0.11	0.25	0.16
2. Gender	−0.11	-	0.08	0.27 **
3. Age	0.25	0.08	-	0.22 *
4. Clinical Ladder	0.17	0.27 **	0.22 *	-

* Correlations are significant (*p* < 0.05) and ** (*p* < 0.01).

**Table 6:** Differences in Resilience Scores at Baseline and Post-intervention.

Variable	Baseline			Post-Intervention		
	**Staff** (*n* = 89)	**NMs** ^1^ (*n* = 8)		**Staff** (*n* = 50)	**NMs** (*n* = 7)	
	*M* (*SD*)	*M* (*SD*)	*p*-value	*M* (*SD*)	*M* (*SD*)	*p*-value
**CD-RISC** ^2^	28.9 (4.8)	27.9 (4.1)	0.65	29.7 (5.0)	40.0 (4.2)	0.88

^1^ Nurse Managers; ^2^ Connor Davidson Resilience Scale.

**Table 7:** Pre-to Post-intervention Resilience Score Changes.

	Pre-Intervention (All)			Pre-Intervention (Dropouts)			Post-Intervention			Post-Preintervention			
	*n*	*M*	*SD*	*n*	*M*	*SD*	*n*	*M*	*SD*	*n*	*M*	*SD*	*p*-Value
Staff	89	28.9	4.8	39	28.3	4.5	50	29.7	5	50	−0.8	4.1	0.19
NMs ^1^	8	27.9	4.1	1	29	N/A	7	30	4.2	7	−2.5	1.5	<0.05
Combined	97	28.7	4.7	40	28.4	4.4	57	29.7	4.9	57	−0.9	3.9	0.07

^1^ Nurse Managers.

**Table 8:** Perceptions of Program Success, Support, Restoration, Implementation.

Study Question	Subcategory	No		Yes		NA	
		*n*	%	*n*	%	*n*	%
**Staff Nurse Evaluation**							
*Did you have leadership or co-worker support to use R2R?*		13	22.8	38	66.7	6	10.5
*Did you feel that the space was appropriate?*		14	27.5	26	50.9	11	21.6
*Did you feel relaxed/restored using the offerings?*	Pocket Guide	3	5.3	24	42.1	30	52.6
	Restorative Movement	2	3.5	13	22.8	42	73.7
	Inspiration	4	7.0	8	14.1	45	78.9
	Meditation	1	1.8	16	28.1	40	70.1
	Soothing Sounds	1	1.8	18	31.6	38	66.6
	Virtual Reality	1	1.8	17	29.8	39	68.4
	Sound Bar	2	3.5	4	7.0	51	89.5
**Nurse Manager Evaluation**							
*Did you feel that the program was successfully implemented?*		2	28.6	5	71.4		

**Table 9:** Ease of Use of Room to Reflect Offerings *.

Component	Ease of Use, *n* (%)				
	Very Easy	Easy	Neither Easy Nor Difficult	Difficult	Not Used
Pocket Guide	23 (40.4)	8 (14.0)	0	0	20 (35.1)
Restorative Movement	4 (7.0)	11 (19.3)	1 (1.8)	0	35 (61.4)
Inspiration	6 (10.5)	9 (15.8)	2 (3.5)	0	34 (59.7)
Meditation	7 (12.3)	12 (21.1)	0	1(1.8)	31 (54.4)
Soothing Sounds	7 (12.3)	8 (14.0)	3 (5.3)	0	33 (57.9)
Virtual Reality	4 (7.0)	9 (15.8)	8 (14.0)	1 (1.8)	28 (49.1)
Sound Bar	2 (3.5)	5 (8.8)	2 (3.5)	0	40 (70.2)

* Percentages reflect those participants who filled out Ease of Use questions.

**Table 10:** Quick Response Video, Audio, Pocket Guide Use.

Format	Offering	Count (%)
Virtual Reality Video	Experience Animals	154 (35)
	Experience Wonder	122 (27.7)
	Experience Nature	120 (27.3)
	Experience Guided Meditation	15 (3.4)
	Experience Travel	29 (6.6)
	**Total VR**	**440**
Other Video	Body Movement	77 (100)
	**Total General Video**	**77**
Audio	Meditation	57 (45.6)
	Inspirational Poems and Quotes	48 (38.4)
	Soothing Sounds	20 (16.0)
	**Total Audio**	**125**
Pocket Guide	Wash	135 (47.4)
	Wait	55 (19.3)
	Wipe	51 (17.9)
	Walk	44 (15.4)
	**Total Pocket Guide**	**285**

## Data Availability

Data are available upon request from the corresponding author. They are not publicly available.
